# ^1^H, ^13^C and ^15^N resonance assignments for human all-Ala α-lactalbumin in its molten globule and urea-denatured states

**DOI:** 10.1007/s12104-026-10260-x

**Published:** 2026-01-23

**Authors:** Lorena Varela, Lorna J. Smith, Christina Redfield

**Affiliations:** 1 Department of Biochemistry, University of Oxford, South Parks Road, Oxford, OX1 3QU UK; 2 School of Biosciences, University of Kent, Canterbury, CT2 7NJ UK; 3 Department of Chemistry, Inorganic Chemistry Lab, University of Oxford, South Parks Road, Oxford, OX1 3QR UK

**Keywords:** α-lactalbumin, NMR resonance assignments, Molten globule state, Urea-denatured state

## Abstract

Molten globules are compact, partially-folded proteins postulated to be general intermediates in protein folding. Human α-lactalbumin (α-LA) is a Ca^2+^-binding, four-disulphide protein whose native structure is divided into two lobes, one is largely helical, the α-domain, and the other has a significant β-sheet content, the β-domain. α-LA forms a “classical” molten globule at low pH which has been studied widely as a model system of a partially-folded protein. The α-LA molten globule is compact and has a native-like helical secondary structure content. All-Ala α-LA, which has all eight native cysteines mutated to alanine, also adopts a partially-folded molten globule conformation and gives a high-quality ^1^H-^15^N HSQC spectrum at pH 2 and 40 °C. The lack of cysteine residues makes all-Ala α-LA a suitable template for spin-labelling studies. In this report we present ^1^H, ^13^C and ^15^N assignments for human all-Ala α-LA in its molten globule and 8 M urea-denatured states. Analysis of the chemical shift data for the molten globule state shows they are consistent with high populations of conformations in the α region of φ,ψ space for residues in the α domain of the protein. In contrast, the data for the urea-denatured state are closely similar to those expected for a random coil.

**Supplementary Information:**

The online version contains supplementary material available at 10.1007/s12104-026-10260-x.

## Biological context

The folding of a protein to its native state from the information contained in its amino acid sequence is a key feature of the conversion of genetic information into biological activity. Although in some cases protein folding has been found to be highly cooperative, in others partially-structured species have been observed to form early in refolding prior to formation of the native state. A variety of studies has shown that such species are compact, have extensive native-like secondary structure but lack the specific side-chain packing characteristic of native structures; these species are frequently known as molten globules. For several proteins, including α-lactalbumin and apo-myoglobin, similar species have been found to be stable at equilibrium under mildly denaturing conditions, such as acidic pH or low concentrations of urea (Arai and Kuwajima 2000; Bhattacharyya and Varadarajan 2013; Bychkova et al. 2018; Hughson et al. 1990; Jennings and Wright 1993; Kuwajima 1996; Ptitsyn 1995). There has been considerable interest in characterising these equilibrium partially-folded molten globule states in detail in order to gain insight into the determinants of the protein fold and of the mechanism of folding.

Human α-lactalbumin (α-LA) is a 14 kDa Ca^2+^-binding protein whose native structure is divided into two domains, one is largely helical (the α-domain) and the other has a significant content of β-sheet (the β-domain) (Acharya et al. 1991). α-LA contains four disulfide bonds, two in the α-domain, C6-C120 and C28-C111, one in the β-domain, C61-C77, and one linking the two domains, C73-C91. The refolding of α-LA from its denatured state is characterised by the rapid formation of a compact species containing a near-native α-helical content but no evidence for tertiary structure (Arai and Kuwajima 1996).

At low pH α-LA undergoes partial unfolding to form a molten globule (Nozaka et al. 1978; Dolgikh et al. 1981). A number of studies have shown that the residual structure in the α-LA molten globule has extensive native-like character. This is particularly evident in the α-domain of the protein where the native-like helices are present and arranged in a manner which bears a similarity to that found in the native structure (Peng et al. 1995; Schulman et al. 1997; Schulman et al. 1995; Song et al. 1998; Wu and Kim 1998; Wu et al. 1995).

The lack of fixed tertiary interactions in molten globules results in a fluctuating ensemble of structures, which interconvert on a millisecond to microsecond time scale giving rise to poor chemical shift dispersion and significant line broadening in the NMR spectrum (Higman et al. 2009; Kim et al. 1999; Redfield 2004); this has made direct study of molten globule species by NMR methods a challenge. Detailed NMR studies of apo-myoglobin at pH 4 and 50 °C have provided residue-specific information for this partially-folded state (Cavagnero et al. 2001; Eliezer et al. 1998). The observed sharpening of NMR resonances as the temperature is increased can be attributed to modifications of the complex µs to ms dynamic properties of the molten globule.

This high-temperature approach has also led to improved ^1^-^15^ N HSQC spectra for the human α-LA molten globule (Ramboarina and Redfield 2003; Ramboarina and Redfield 2008). However, the presence of four disulfide bonds in α-LA leads to significant line broadening for residues in the vicinity of the cysteines even at 50 °C. A variant of human α-LA, in which all eight cysteines have been replaced by alanine (all-Ala α-LA), also forms a compact and helical molten globule at low pH, demonstrating that the overall architecture of the α-LA molten globule fold is determined by the polypeptide sequence, and not as the result of cross-linking by disulphide bonds (Peng et al. 1995; Redfield et al. 1999). At 40 °C, all-Ala α-LA, gives a high-quality ^1^H-^15^N HSQC spectrum in which peaks from all residues can be identified. The absence of disulphide bonds in all-Ala α-LA also makes it a suitable template for the introduction of spin-labelled cysteines for determination of tertiary structural information using DEER experiments. In this report we present and analyse ^1^H, ^13^C and ^15^N assignments for human all-Ala α-LA in its molten globule and 8 M urea-denatured states. These data provide the starting point for the definition of the structural ensemble that describes the partially-folded molten globule state.

## Methods and experiments

### Protein expression and purification

Human α-lactalbumin with its eight cysteine residues replaced by alanines (all-Ala α-LA) was expressed in BL21(DE3) cells (Peng et al. 1995). The expression vector (pET-30) contained an N-terminal His_6_ tag for purification with a TEV protease cleavage site. The TEV cleavage left an extra N-terminal glycine (G0). ^15^N and ^15^N/^13^C labelled protein was produced by initially growing cells in LB medium to boost the rate of cell growth. 2 mL of starter culture, grown in LB at 37 °C for ∼15 h, was added to 1 L of medium containing 100 µg/mL kanamycin. Cells were grown at 37 °C to an OD_600_ ∼ 0.8 and then collected by centrifugation (~ 9000 g, 4 °C), washed with M9 salts buffer and resuspended into M9 minimal medium (4 L LB/1 L M9 ratio) containing 1 g/L ^15^NH_4_Cl and 4 g/L glucose or ^13^C-glucose, for ^15^N or ^15^N/^13^C labelled protein, respectively. Cells were then incubated at 30 °C for an hour (to allow them to adapt to their new conditions). Expression was induced with isopropyl-β-D-thiogalactopyranoside (IPTG) at a final concentration of 1 mM. Cells were grown at 30 °C for at least 12 h prior to being spun down (~ 9000 g, 4 °C) and then resuspended into 35 mL of 50 mM TRIS buffer at pH 8.0 containing 150 mM NaCl, 6 µL/mL of a 2.5 mg/mL DNase stock solution, 1.2 mg/mL of hen egg white lysozyme and one protease inhibitor cocktail tablet. The solution was incubated at 4 °C for 30 min. The cells were then disrupted using a French pressure cell (1000 psi) and the cell lysate was spun down (25000 g, 4 °C). The inclusion body pellet was solubilised into 40 mL of denaturing buffer (50 mM TRIS, 150 mM NaCl, 6 M GuHCl, pH 8.0) with 0.5% v/v Triton X100, and stirred at room temperature for 30 min. The solution was finally ultra-centrifuged (~ 256600 g, 4 °C). The supernatant was loaded onto a Ni^2+^ Fast Flow Chelating Sepharose column (Amersham Biosciences). The protein bound to the column was washed with buffer containing decreasing concentrations of GuHCl and then eluted with 50 mM EDTA solution at pH 8.0. The His_6_ tag was cleaved by adding 0.2 mg/mL of TEV protease and 5 mM DTT to the protein elution fraction followed by dialysis against 2 L of TEV reaction buffer at room temperature for 15 hours in the dark. All traces of EDTA were then removed by dialysis against 50 mM TRIS buffer at pH 8.0. The protein solution was then denatured in 6 M GuHCl, 50 mM TRIS buffer at pH 8.8 and loaded again onto a Ni^2+^ Fast Flow Chelating Sepharose column to separate the mixture of cleaved and uncleaved protein as well as TEV protease. Purified denatured protein was acidified to pH 2 and dialysed against water. The pure protein was then lyophilized.

### NMR spectroscopy

Experiments for resonance assignment were performed using ^15^N- or ^15^N/^13^C-labelled all-Ala α-LA. NMR experiments were carried out on three different spectrometers: a home-built spectrometer with ^1^H-operating frequency of 750 MHz, a triple-resonance probe and GE/Omega console, and Bruker Avance 500 and 750 MHz spectrometers equipped with TCI CryoProbes. Backbone ^1^H^N^ and ^15^N assignments for the all-Ala α-LA molten globule at 40 °C and the urea-denatured state at 20 °C had been obtained previously using data from 3D ^15^N-edited TOCSY-HSQC, NOESY-HSQC and HSQC-NOESY-HSQC experiments acquired using ^15^N-labelled protein (Ramboarina and Redfield 2008; Redfield et al. 1999). Backbone assignments were confirmed and extended for the molten globule state using ^15^N/^13^C-labelled all-Ala α-LA at a concentration of 0.3 mM in 95% H_2_O/5% D_2_O (v/v) at pH 2. HNCA, CBCANH, CBCA(CO)NH, HNCO, HN(CA)CO and HBHA(CBCACO)NH experiments were acquired at 40 °C. For the urea-denatured state, HNCA, CBCA(CO)NH, HNCO, HN(CA)CO, HN(CO)CA, HBHA(CBCACO)NH experiments were acquired at 20 °C using ^15^N/^13^C-double labelled all-Ala α-LA at a concentration of 0.3 mM in 8 M urea in 95% H_2_O/5% D_2_O (v/v) at pH 2. Side-chain assignments for both samples were obtained using 3D H(CCCO)NH, (H)CC(CO)NH, HCCH-TOCSY, 3D ^13^C-edited NOESY-HSQC and 2D ^1^H-^13^ C HSQC experiments. NMR data were processed using NMRPipe (Delaglio et al. 1995) and analysed using CcpNmr Analysis (Vranken et al. 2005). ^1^H chemical shifts were referenced using the H_2_O peak (4.60 ppm at pH 2 at 40 °C and 5.04 ppm in 8 M urea at 20 °C), previously calibrated with DSS, and ^13^C and ^15^N were referenced indirectly.

### MERA calculations

Populations of the α and β regions of φ,ψ space for both the molten globule and urea-denatured states were calculated using output from the web server MERA (Maximum Entropy Ramachandran map Analysis from NMR data) (https://spin.niddk.nih.gov/bax-apps/nmrserver/mera/) (Mantsyzov et al. 2014, 2015). The α and β regions of φ,ψ space are defined as given in Smith et al. (1996). Backbone ^13^Cα, ^13^CO and ^15^N chemical shifts, ^3^J_HNHα_ coupling constant values from 2D HMQCJ spectra, and cross-relaxation rates from intra-residue αH-NH and sequential αH-NH and NH-NH NOEs were used as input for MERA. The ^15^N relaxation derived J(0) spectral densities were also used for the urea state calculations (Higman et al. 2009). The calculations were performed for 30 × 30 voxels using the same uncertainty parameters σ(q) for both the urea-denatured and molten globule states. In particular, σ(q) was 1.0 ppm for ^13^Cα and ^13^CO, 2.5 ppm for ^15^N, 0.6 Hz for ^3^J_HNHα_ and 15% of the measured rate plus 15% of the rate predicted from the intra-residue d_HNHα_(i, i) for the cross-relaxation rates. Calculations were performed using a local diffusion anisotropy value of 1.0 and θ values of 0.0, 0.1, 0.2, 0.4, 0.8, 1.6, 3, 6 and 10. Data calculated with a θ value of 0.4 was used for further analysis, with results for residues with a χ^2^ value greater than 1.5 being excluded. In addition, for the urea-denatured state a θ value of 500 was used in MERA calculations to provide a prediction for the populations in the random coil state.

## Extent of assignments and data deposition

**Fig. 1:**
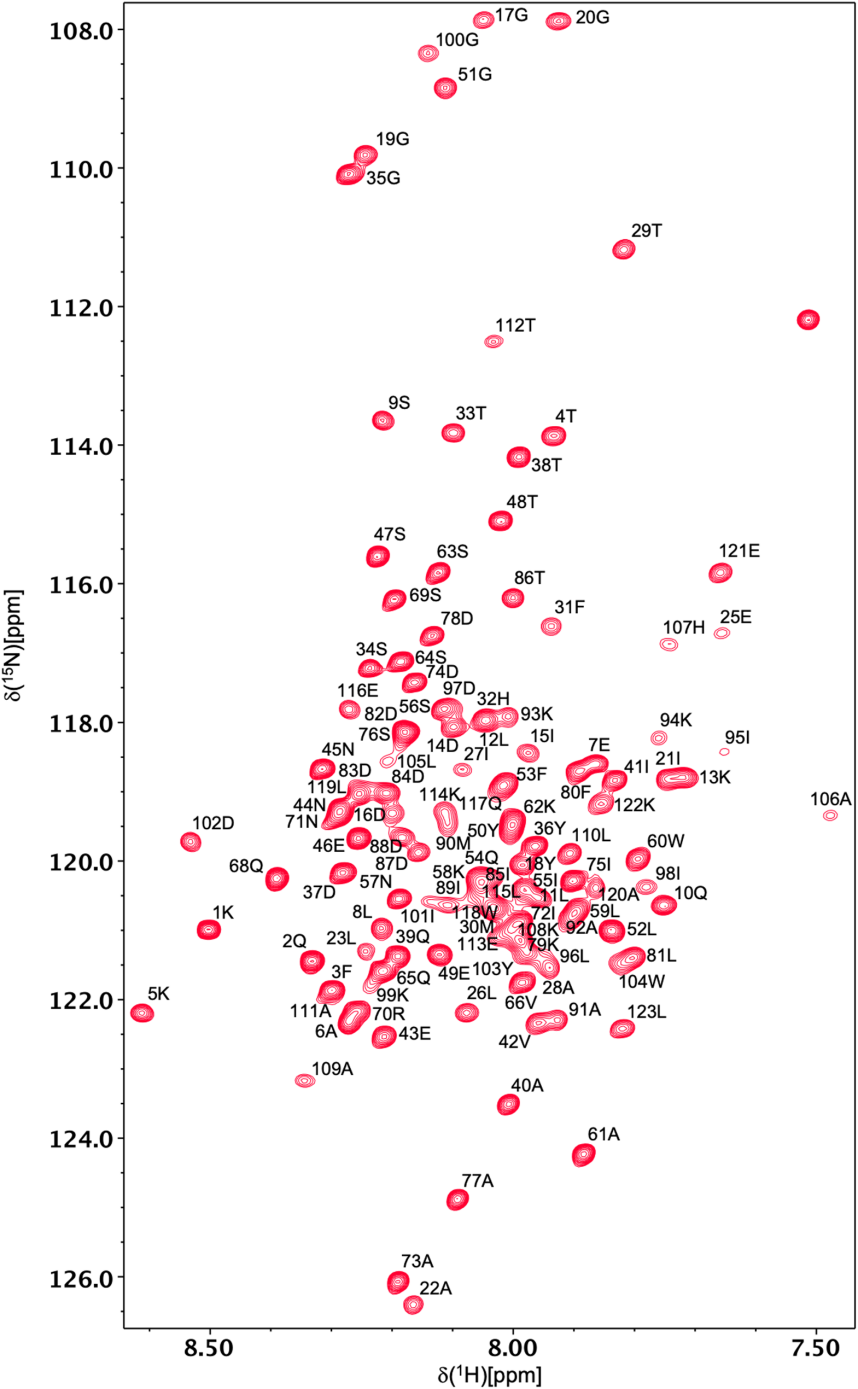
500 MHz ^1^H-^15^N HSQC spectrum of the all-Ala α-LA molten globule at pH 2, 40 °C, in 95% H_2_O/5% D_2_O. Peak assignments for backbone amides of residues 1–123 are indicated in black

**Fig. 2:**
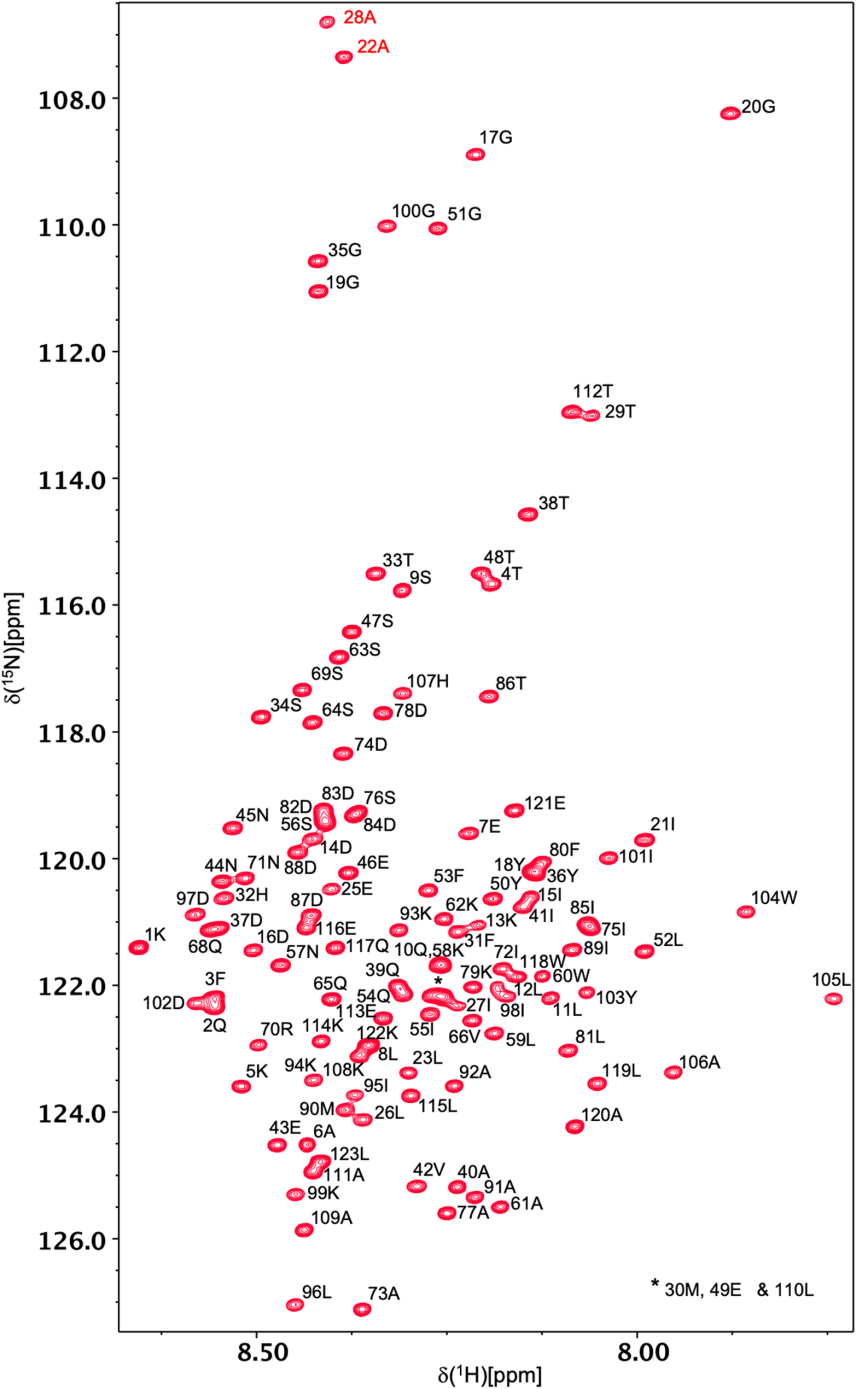
500 MHz ^1^H-^15^N HSQC spectrum of denatured all-Ala α-LA at pH 2 in 8 M urea, 20 °C, in 95% H_2_O/5% D_2_O. Peak assignments for backbone amides of residues 1–123 are indicated in black. The peaks corresponding to A22 and A28, labelled in red, are folded in the ^15^N dimension; their correct ^15^N chemical shifts are 128.4 and 127.8 ppm, respectively

Backbone ^1^H^N^ and ^15^N resonances have been assigned for all 121 non-proline residues of all-Ala α-LA in both the molten globule and urea-denatured states at pH 2 (Figs. 1 and 2). ^1^Hα, ^1^Hβ, ^13^Cα, ^13^Cβ and ^13^CO assignments were also obtained for all 123 residues in both states. A high level of side-chain assignment was also achieved for both states of the protein. For the molten globule, 89%, 89%, 64%, 64%, 21% and 18% of ^1^Hγ, ^13^Cγ, ^1^Hδ, ^13^Cδ, ^1^Hε, ^13^Cε assignments were obtained, respectively. The lower level of assignment achieved at the δ and ε positions reflects the absence of peaks for these resonances in the H(CCCO)NH and (H)CC(CO)NH spectra. For the urea-denatured α-LA, 85%, 98%, 66%, 75%, 21% and 33% of ^1^Hγ, ^13^Cγ, ^1^Hδ, ^13^Cδ, ^1^Hε, ^13^Cε assignments were obtained, respectively. The chemical shift assignments for both states have been deposited in the BioMagResBank (http://www.bmrb.wisc.edu) under accession codes 26747 (for the all-Ala α-LA molten globule) and 26749 (for the all-Ala α-LA urea-denatured state).

**Fig. 3:**
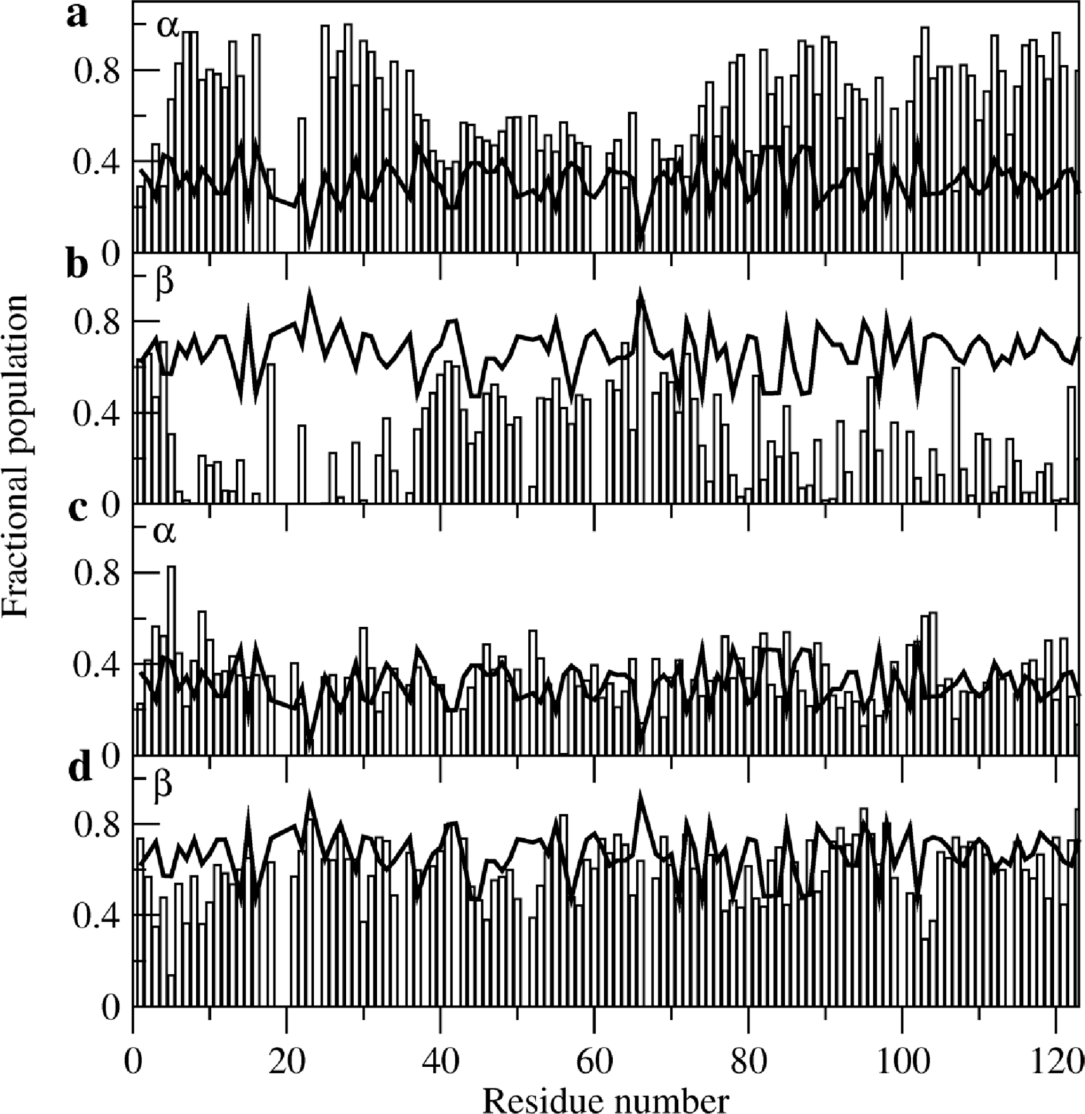
The populations of the α and β regions of Φ·Ψ space for the molten globule (panels a and b) and urea-denatured states (panels c and d) calculated from backbone chemical shift, ^3^J_HNHα_ coupling constant and cross-relaxation rate data using the MERA webserver (Mantsyzov et al. 2014, 2015). The predicted populations for a random coil are shown by a solid line.

The chemical shift data for all-Ala α-LA were analysed using the MERA webserver. (Mantsyzov et al. 2014, 2015) This approach, which was developed particularly for disordered proteins, gives backbone φ,ψ distributions for individual residues in the protein based on experimental NMR data (chemical shifts, coupling constants and cross-relaxation rates from intra-residue and sequential NOEs). Example φ,ψ distributions for R70 and T86 in both the molten globule and urea-denatured states of all-Ala α-LA are shown in Supplementary Material (Figure S1). To gain an overview of the results, the populations of the α and β regions of φ,ψ space were calculated for each residue from the MERA output Ramachandran plot distributions and were compared with the populations predicted for a random coil (Fig. 3). For the urea-denatured state the α and β populations predicted for all-Ala α-LA are closely similar to those predicted for a random coil throughout the protein sequence (Fig. 3c-d). However, for the molten globule state residues 5–16, 25–37, 74–77, 77–79, 82–84, 86–91, 93–94, 97–106, 108–109, 111–113, 115–121 and 123 have α region populations greater than 0.6, significantly higher than those predicted for a random coil (Fig. 3a-b). Only for residues 1–2 and 4 at the N-terminus and residues 41–42 and 64, 66 and 72 in the β-domain are there groups of residues with β populations greater than 0.6. Indeed, for only a few residues at the N-terminus and in the β-domain of the protein are the overall populations for the molten globule state similar to those predicted for a random coil. These results are in accord with previous studies that show that in the molten globule state the α-domain contains native-like helical structure and restricted backbone mobility (Peng et al. 1995; Ramboarina and Redfield 2008; Schulman et al. 1995). The NMR assignments and structural propensity predictions from MERA provide the starting point for the definition of the structural ensemble that describes the partially-folded molten globule state of all-Ala α-LA.

## Supplementary Information

One figure comparing the φ,ψ distribution calculated using MERA for R70 and T86 in the molten globule and urea-denatured states of all-Ala α-LA.

## Supplementary Information

Below is the link to the electronic supplementary material.

Supplementary Material 1

## Data Availability

Assignments for human all-Ala α-lactalbumin in its molten globule and urea-denatured states have been deposited in the BMRB under accession numbers 26747 and 26749, respectively.
