# Supplementary material for: 1H, 13C and 15N resonance assignments for human all-Ala α-lactalbumin in its molten globule and urea-denatured states
Source: Biomol NMR Assign. 2026 Jan 23;20(1):11. doi: 10.1007/s12104-026-10260-x (PMC12827368; doi:10.1007/s12104-026-10260-x)
Supplement: Supplementary file 1 — Supplementary Material 1 [file 12104_2026_10260_MOESM1_ESM.pdf]

Supplementary Material for ^{1}H, ^{13}C and ^{15}N resonance assignments for human all-Ala α-lactalbumin in its molten globule and urea-denatured states. Lorena Varela, Lorna J. Smith and Christina Redfield*

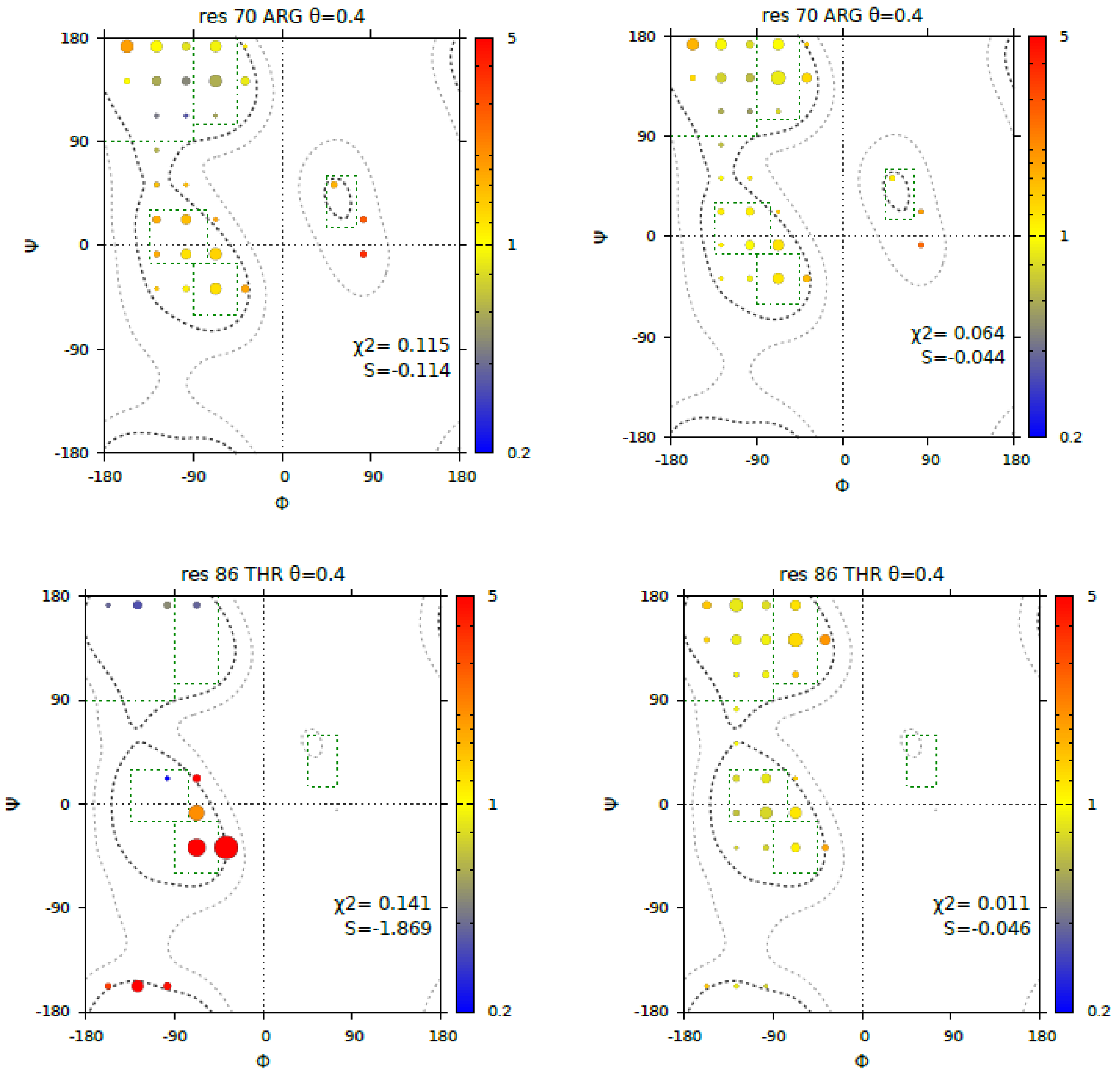

Figure S1

Comparison of the Ramachandran ϕ,ψ plots for Arg 70 (β-domain) and Thr 86 (α-domain) calculated using MERA with θ=0.4. The left-hand panels are for the molten globule state and the right-hand panels are for the urea denatured state. The ϕ,ψ populations are similar for Arg 70 in the molten globule and urea-denatured state but Thr 86 shows a much higher population of the α-region in the molten globule compared to the urea-denatured state.
